# BAlloon versus Stenting in severe Ischaemia of the Leg-3 (BASIL-3): study protocol for a randomised controlled trial

**DOI:** 10.1186/s13063-017-1968-6

**Published:** 2017-05-19

**Authors:** Benjamin D. Hunt, Matthew A. Popplewell, Huw Davies, Lewis Meecham, Hugh Jarrett, Gareth Bate, Margaret Grant, Smitaa Patel, Catherine Hewitt, Lazaros Andronis, Jonathan J. Deeks, Andrew Bradbury, on behalf of the BASIL-3 Collaborative Group

**Affiliations:** 1 Birmingham Clinical Trials Unit, Institute of Applied Health Research, College of Medical and Dental Sciences, Public Health Building, University of Birmingham, Birmingham, B15 2TT UK; 2 Heart of England NHS Foundation Trust, Netherwood House, Solihull Hospital, University Department of Vascular Surgery, Lode Lane, Solihull, B91 2JL UK; 3 Health Economics Unit, Institute of Applied Health Research, Public Health Building, University of Birmingham, Birmingham, B15 2TT UK

**Keywords:** Severe limb ischaemia, Critical limb ischaemia, Endovascular treatment, Angioplasty, Stent, Drug-coated balloon, Drug-eluting stent, Diabetes, Cost-effectiveness

## Abstract

**Background:**

Severe limb ischaemia (SLI) is defined as the presence of rest pain and/or tissue loss secondary to lower extremity atherosclerotic peripheral arterial disease. The superficial femoral and popliteal arteries are the most commonly diseased vessels in such patients and are being increasingly treated using endovascular revascularisation techniques. However, it is currently unknown whether drug-eluting stents and drug-coated balloons confer additional clinical benefits over more established techniques using plain balloons and bare metal stents, or whether they represent a cost-effective use of NHS resources.

**Methods:**

The BASIL-3 trial is a UK National Institute for Health Research, Health Technology Assessment Programme-funded, multicentre, randomised controlled trial (RCT) comparing the clinical and cost-effectiveness of plain balloon angioplasty with or without bail-out bare metal stenting, drug-coated balloon angioplasty with or without bail-out bare metal stenting, and primary stenting with drug-eluting stents for SLI secondary to femoro-popliteal disease. Patients with ‘multilevel’ disease may receive aorto-iliac and/or infrapopliteal treatments concurrently with their randomised femoro-popliteal intervention. The primary clinical outcome is amputation-free survival defined as the time to major (above the ankle) amputation of the index limb or death from any cause. The primary outcome for the economic analysis is cost per quality-adjusted life year. Secondary outcome measures include overall survival, major adverse limb events, major adverse cardiac events, relief of ischaemic pain, healing of tissue loss, and quality of life. The required sample size has been calculated at 861 participants (287 on each arm). These patients will be recruited over 3 years and followed-up for between 2 and 5 years.

**Discussion:**

BASIL-3 is a pragmatic RCT designed to reflect current UK clinical practice. The results will inform decision-making regarding the appropriateness of funding the use of drug-coated balloons and drug-eluting stents, by the NHS, for the management of SLI due to femoro-popliteal disease.

**Trial registration:**

ISRCTN Registry, identifier: ISRCTN14469736. Registered on 22 October 2015.

**Electronic supplementary material:**

The online version of this article (doi:10.1186/s13063-017-1968-6) contains supplementary material, which is available to authorized users.

## Background

Peripheral arterial disease (PAD) is caused by a gradual build-up of atheroma within the arterial wall leading to the lumen becoming stenosed (narrowed) and ultimately occluded. Modifiable risk factors for the development of PAD include cigarette smoking, hypertension, dyslipidaemia, diabetes mellitus (DM), chronic kidney disease (CKD), increased Body Mass Index, and sedentary lifestyle. PAD is also associated with increasing age and male sex, and there may be a familial predisposition in some cases [1]. PAD presents as a continuum from asymptomatic disease to intermittent claudication (pain in the leg on walking due to ischaemia, relieved by rest) through to severe (or critical) limb ischaemia (SLI) characterised by [2]:

1. Severe and constant ischaemic pain which is experienced at rest, especially at night, usually in the forefoot
2. Tissue loss in the form of ulceration or gangrene, typically this starts with a minor foot injury which fails to heal and/or becomes infected

In the Western world, it has been estimated that there will be between 500 and 1000 new cases of SLI each year per million general population although the current epidemiology of SLI in the United Kingdom (UK) is not well defined [3]. The incidence of SLI is increasing globally as a consequence of ageing populations and the increasing prevalence of modifiable risk factors, especially tobacco consumption and DM [4]. SLI patients tend to have multiple comorbidities, to be at high cardiovascular risk, and untreated have up to 50% mortality within 1 year of diagnosis [5]. Failure to promptly revascularise in SLI patients is associated with greater use of health care services and poorer outcomes including major amputation and death [6].

SLI is currently managed by the following methods [5, 6]:

1. Best medical therapy, comprising antiplatelet medication, lipid modification, optimal diabetic/blood pressure control, analgesia, and foot and wound care
2. Surgical revascularisation, usually with autologous vein bypass and/or endarterectomy
3. Endovascular revascularisation, performed under local anaesthetic and with balloon catheters and/or stents
4. Primary amputation, where the limb is considered to be unsalvageable or the patient is either unwilling or unable to undergo revascularisation
5. End of life care for those deemed unfit for revascularisation or primary amputation

Endovascular revascularisation techniques are increasingly being used as a first-line treatment for SLI, especially when a suitable vein is not available for bypass. Traditionally, best endovascular treatment consists of plain balloon angioplasty (PBA) with so-called ‘bail-out’ bare metal stenting (BMS) when PBA alone has been technically unsuccessful. More recently, drug-coated balloons (DCB) and drug-eluting stents (DES) have entered the market. These devices are designed to deliver various drugs (most commonly paclitaxel) locally to the lesion with the aim of reducing the incidence of restenosis following angioplasty or stenting [7, 8]. However, evidence of clinical efficacy is lacking and there are unknown NHS cost implications for their use when compared with PBA and BMS. Consequently, the National Institute for Health and Care Excellence (NICE) and the National Institute for Health Research’s Health Technology Assessment (HTA) programme both recommended that a randomised controlled trial (RCT) be conducted in patients with SLI to establish whether these new drug technologies confer additional clinical benefit and represent a cost-effective use of the available NHS resources [5, 9].

**Fig. 1:**
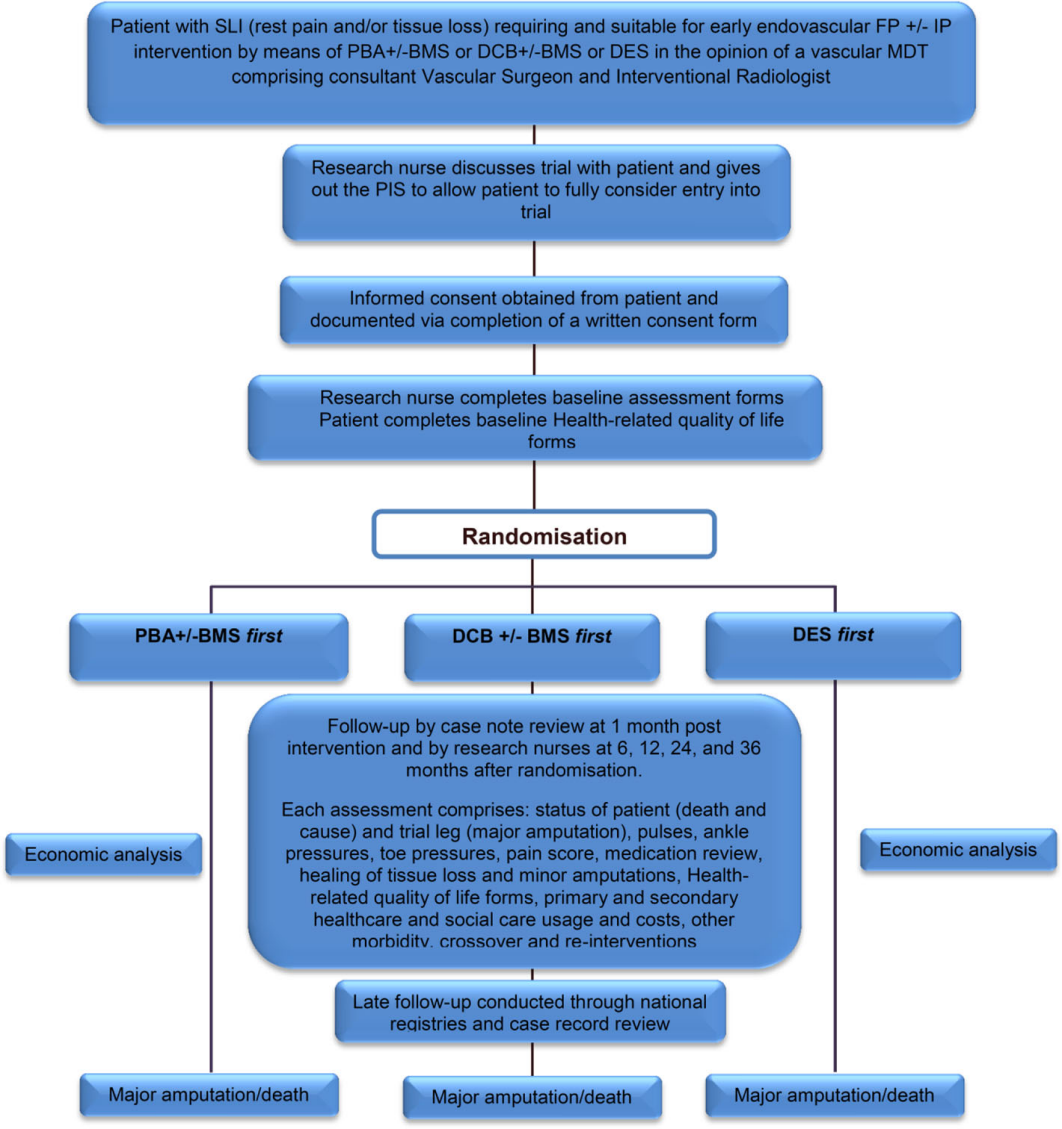
Flow diagram of study design

**Fig. 2:**
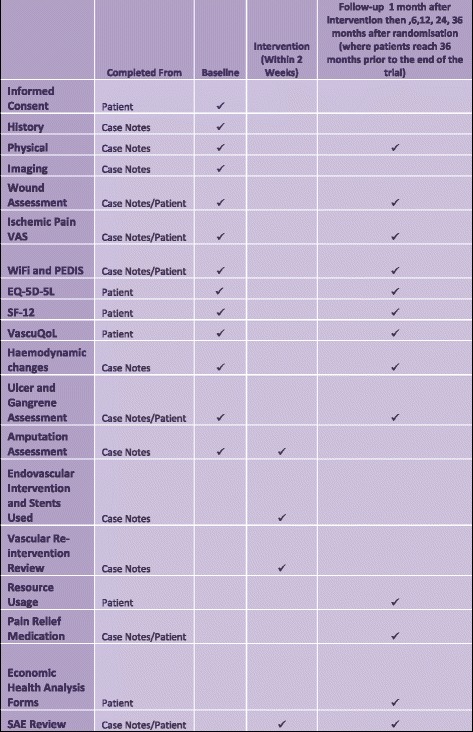
Standard Protocol items: Recommendations for Interventional Trials (SPIRIT) diagram

The BASIL-3 trial directly addresses the HTA call (13/81) as a RCT in which patients with SLI secondary to atherosclerotic femoro-popliteal (FP) disease ± infrapopliteal (IP) disease are randomly allocated to: PBA ± bail-out BMS, DCB ± bail-out BMS, or primary DES as their first revascularisation strategy (see Figs. 1 and 2 for the BASIL-3 Consolidated Standards of Reporting Trials (CONSORT) and Standard Protocol Items: Recommendations for Interventional Trials (SPIRIT) diagrams, respectively. A SPIRIT Checklist is also provided as Additional file 1). BASIL-3 includes an internal pilot phase and health economic analysis. The primary clinical outcome is amputation-free survival (AFS) defined as the time to major (above the ankle) amputation of the index limb or death from any cause, whichever occurs first; secondary outcome measures are shown in Table 1. The primary outcome for the cost-effectiveness analysis is cost per quality-adjusted life year (QALY) gained.

**Table 1:** Secondary outcome measures

Overall survival	
Major adverse limb events (MALE), defined as amputation (transtibial or above) or any major vascular re-intervention (thrombectomy, thrombolysis, PBA, stenting, or surgery)	
In-hospital and 30-day morbidity and mortality	
Major adverse cardiac events (MACE), defined as SLI and amputation affecting the contralateral limb, ACS, or stroke	
Relief of ischaemic pain (VAS, medication usage)	
Psychological morbidity (using HADS)	
Quality of life using generic (EQ-5D-5L, ICECAP-O, SF-12) and disease-specific (VascuQoL) tools	
Re- and cross-over intervention rates	
Healing of tissue loss (ulcers, gangrene) as assessed by the PEDIS and the WIFI scoring and classification systems	
Extent and healing of minor (toe and forefoot) amputations (also using PEDIS and WIFI)	
Haemodynamic changes; absolute ankle and toe pressures, ABPI, TBPI	

*ABPI* ankle-brachial pressure index, *PBA* plain balloon angioplasty, *EQ-5D-5L* European quality of life 5 level score, *HADS* Hospital Anxiety and Depression Scale, *ICECAP-O* ICEpop CAPability measure for Older people, *PEDIS* Perfusion Extent Depth Ischaemia Sensation, *SF-12* Short form-12 health survey version 2, *TBPI* toe-brachial pressure index, *VAS* Visual Analogue Scale, *WIFI* Wound, Ischaemia, and Foot Infection tool

## Methods

### Indemnity

This is a clinician-initiated study. The sponsor (University of Birmingham) holds public liability (negligent harm) and clinical trial (negligent harm) insurance policies which apply to this trial. Full details of indemnity arrangements for this trial can be obtained from the sponsor.

### Eligibility

Full inclusion/exclusion criteria are shown in Table 2. All patients with SLI referred to vascular units within participating NHS organisations may be considered for enrolment (a full list of participating centres can be obtained from the trial sponsor). Participants should have atherosclerotic FP disease and be judged by the vascular multidisciplinary team (consisting of two endovascular practitioners from either vascular surgery or interventional radiology) to require early endovascular revascularisation. Following diagnostic imaging, all participants should be deemed suitable to receive any of the three trial revascularisation strategies and should also have adequate inflow to support these strategies (either at the time of randomisation or achieved as part of a ‘hybrid’ procedure in which inflow is restored concurrently with the FP revascularisation). Participants should have an anticipated life expectancy of at least 6 months and be able to complete the trial quality of life (QoL) and resource use questionnaires. Patients will be excluded from the trial if they lack capacity to provide written informed consent, have received a previous intervention to the target vessel within the past 12 months, or speak insufficient English to guarantee informed consent where translation services are not available.

**Table 2:** Inclusion and exclusion criteria

Inclusion criteria	Exclusion criteria
Have SLI due to atherosclerotic FP, ± IP, PAD	Have an anticipated life expectancy <6 months
Be judged by the responsible clinicians (consultant VS, IR) working as part of a MDT to: Require early FP, ± IP, endovascular revascularisation in addition to BMT, foot and wound care	Be, in the opinion of the clinician, unable to provide informed consent
Have or will have adequate ‘inflow’ to support all trial revascularisation strategies	Be a non-English speaker where local translation facilities are insufficient to guarantee informed consent
Judged suitable for all trial revascularisation strategies following diagnostic imaging and a documented MDT discussion	Be judged unsuitable for the endovascular revascularisation strategies by a vascular MDT
Able to complete QoL and resource use booklet	Previous intervention (BET or bypass) to the target vessel within the past 12 months

*BET* best endovascular therapy *BMT* best medical therapy *IP* infrapopliteal *FP* femoro-popliteal *MDT* multi-disciplinary team *PAD* peripheral arterial disease, *QoL* quality of life *SLI* severe limb ischaemia

### Randomisation

Randomisation will be provided by a computer-generated programme hosted online from Birmingham Clinical Trials Unit (BCTU), using a minimisation algorithm with a random element incorporated within it to ensure balance between the three arms with regard to important clinical variables. The following minimisation variables will be used:

- Age (≤60, 61–70, 71–80, or >80 years)
- Sex (male or female)
- DM (presence or absence)
- CKD (presence or absence of stage 3 or higher)
- Severity of clinical disease (ischaemic rest pain only, tissue loss only, or both)
- Artery being treated (superficial femoral, popliteal, or both)
- Recruiting clinical centre
- Prior permissible intervention to the trial leg, defined as an intervention to the target vessel more than 12 months ago or to a different vessel in the trial leg at any time (yes or no)
- Hybrid procedure planned (yes or no)

Randomisation will be performed between the three endovascular interventions at a ratio of 1:1:1.

### Baseline assessment

All participants will undergo a baseline assessment consisting of:

1. Patient’s medical history including cardiovascular risk factors, comorbidities, functional status as well as prior vascular interventions and amputations to both legs
2. Clinical status comprising peripheral pulses, ankle-brachial pressure index (ABPI), toe-brachial pressure index ((TBPI) where indicated), and current drug therapy
3. Foot assessment using the ‘Wound, Ischaemia, and Foot Infection’ (WiFi) and ‘Perfusion Extent Depth Ischaemia Sensation’ (PEDIS) tools [10, 11]
4. Review of investigations including diagnostic imaging.
5. Assessment of patient’s QoL by use of the following tools:
  - European quality of life 5 level score (EQ-5D-5L) [12]
  - ICEpop CAPability measure for Older people (ICECAP-O) [13]
  - Short form-12 health survey version 2 (SF-12) [14]
  - Visual Analogue Scale pain score (VAS) [15]
  - Hospital Anxiety and Depression Scale (HADS) [16]
  - Vascular quality of life (VascuQoL) [17]

### Trial intervention

The randomised trial intervention should normally take place within 14 days of randomisation where clinically and logistically practical. The randomised endovascular procedure will normally be carried out under local anaesthesia with access via the common femoral artery. Technical success of the procedure will be assessed by pulse status, completion angiography, and haemodynamic measures such as ABPI. Data collected relating to the randomised procedure will include type and number of the devices used by artery segment as well as disease severity. Standard data relating to the hospital admission or day case will be collected and include summary of the admission, discharge destination, additional interventions, and medical complications.

### Repeat and cross-over interventions

Further intervention is possible in all arms of the trial, even when the trial endovascular intervention has been successful (for example, due to multilevel disease or following restenosis). This may either be with the same endovascular intervention (re-intervention), one of the alternative endovascular interventions (endovascular cross-over intervention) or surgical intervention (surgical cross-over intervention), each of which may be repeated more than once.

Based on clinical experience, and data from the original BASIL trial [18], we anticipate that further intervention:

- Will be required in up to 20% of participants
- Is most likely to be required within 12 months of randomisation

### Follow-up

Outcomes will be recorded at 1 month post intervention and 6, 12, 24, and 36 months post randomisation. Data collected will include peripheral pulses, ABPI, TBPI (where indicated), functional status, and current drug therapy. All participants will undergo a full review of hospitalisations, further interventions (vascular, nonvascular, and amputations), and (serious) adverse events (SAEs/AEs). The QoL assessments (previously described) will be completed at each follow-up time point as well as health resource usage and personal circumstance assessments as part of the economic analysis. A schedule of assessments for all time points is given in Fig. 2.

Appropriate strategies to promote participant retention will be considered and implemented by the Trial Management Group (TMG) as required.

Outcomes will be collected by research nurses at the clinical centres on paper Case Report Forms (CRFs). All data will be handled in accordance with the UK Data Protection Act, 1998. CRFs, other than the Patient Contact Form and Consent Form, will not bear the participant’s name. For all other forms the participant’s initials, date of birth and trial number will be used for identification.

### Safety reporting

The collection and reporting of AEs and SAEs will be in accordance with Good Clinical Practice (GCP) and the Research Governance Framework 2005. Safety will be assessed continuously throughout the trial. There are no investigational medicinal products being used as part of BASIL-3 and all of the surgical procedures being tested in this trial are part of current UK ‘standard of care’; therefore, no (S)AEs are anticipated as a unique consequence of participation in BASIL-3. In addition, at regular time points, the Trial Steering Committee (TSC) and Data Monitoring Committee (DMC) will be provided with details of all SAEs.

### Sample size calculation

The sample size for this trial was calculated based on a time-to-event analysis making two key comparisons between standard care and the new treatments (PBA ± BMS versus DES; and PBA ± BMS versus DCB ± BMS). To maintain an overall 5% type I error rate, each comparison will be tested at a significance level of 2.5% to account for the increase in the risk of type I error associated with making two key comparisons. The total trial duration is 5 years with 3 years’ recruitment (20% of participants are to be recruited in year 1, and 40% in years 2 and 3) and 2 years’ follow-up resulting in a mean follow-up of 3.3 years per patient.

The sample size calculation is based on estimated event rates in the PBA ± BMS arm taken from the angioplasty arm of the original BASIL trial (observed to be 0.70, 0.64, 0.52, 0.46, and 0.36 at the end of years 1–5, respectively) [18]. The study is powered at 90% to detect a hazard ratio of 0.60 for both comparisons reducing the risk of the primary outcome (AFS). Across the three arms, a total of 342 events would be required to detect a hazard ratio of 0.60 (equivalent to an absolute difference in AFS of 13% at year 2) at the 2.5% significance level. Conservatively, allowing for 5% drop out for the primary outcome (equivalent to 1% dropout in each year for 5 years) a total of 861 participants is required. It is anticipated that around 50 UK clinical centres will be required to achieve this recruitment target. Appropriate strategies to meet recruitment targets will be discussed and implemented by the TMG as required.

### Pilot phase

Recruitment to BASIL-3 will be reviewed at the end of a 12-month pilot phase and discontinuation of the trial will be considered if:

- Fewer than 15 clinical centres have been opened
- Fewer than two out of three opened centres have recruited a participant
- Fewer than 100 participants have been randomised
- Fewer than 80% of participants have received their allocated treatment

The completeness of the QoL measures will also be evaluated at the end of the pilot phase. If completion rates are poor, consideration will be given to discontinuing some of these tools.

### Data monitoring and interim analysis

After the first year of recruitment, we aim to assess recruitment, retention, patient burden, and completeness of data. A full efficacy and safety analysis report will be reviewed by the DMC on an annual basis or more frequently if required by the DMC or TMG. The DMC will outline and agree the stopping rules for the trial which will be documented in the DMC charter. It is likely that the Haybittle-Peto boundary will be used. This approach states that if an interim analysis of the primary outcome shows, with a *p* value of less than 0.001, that the treatments are different, then the trial should be stopped early. This Haybittle-Peto approach will be used as stopping guide, alongside data on important secondary endpoints and all other relevant evidence. A DMC report and charter outlining the terms of reference (including information on stopping rules) will be agreed with the DMC. The report will specify which endpoints are to be included in the reports to the TSC. The membership of the DMC is stated within the current trial protocol which is published online [19].

Monitoring of BASIL-3 will ensure compliance with GCP. A risk proportionate approach to the initiation, management and monitoring of BASIL-3 will be adopted. Clinical centres will permit trial-related monitoring, audits, and Main Research Ethics Committee review; providing direct access to source data/documents. Trial participants will be informed of this during the informed consent discussion and will consent to provide access to their medical notes for these purposes. Important changes to the trial conduct or trial protocol will be communicated to all relevant interested parties and the trial sponsor assumes responsibility for this.

### Final analysis

The final analysis for the BASIL-3 trial will occur once the last randomised patient reaches the 24-month follow-up assessment. Differences in the primary outcome (AFS) will be assessed by comparing time from randomisation to major limb amputation or death from any cause between randomised groups, assessed up until the end of the follow-up period which will be between 24 and 60 months.

The primary unadjusted analysis will use Kaplan-Meier plots and test the difference between groups using the log-rank test. Data will be censored when individuals reach the end of follow-up or are lost to follow-up prior to reaching the primary outcome. Further analysis of the primary outcome will involve fitting flexible parametric survival models to estimate both the relative and absolute differences in the hazard of the primary outcome, to model the underlying differences in hazard, and to allow for nonproportional hazards. Addition of covariates to this model will allow adjustment for any baseline differences, and the addition of their interactions with the treatment allocation variable will test for subgroup effects. These models will allow examination of differences in effect for short-, medium-, and longer-term follow-up. The primary analysis of AFS will be undertaken on an intention-to-treat basis according to allocated first intervention, regardless of whether the intervention was delivered and whether repeat and cross-over interventions were subsequently undertaken.

Secondary outcome measures that are based on a continuous scale will be analysed using a repeated measures, multilevel model to examine any differential effect over time. Where necessary, data transformations will be made to fulfil modelling assumptions. Treatment effects from the repeated measures model will be reported at the 1-month, 12-month, and ‘end of follow-up’ time points.

Other outcome measures will be explored using standard methods (Fisher’s Exact Test for dichotomous outcomes, log-rank test for time to event data) and will also be reported at 1 month, 12 months, and at the end of follow-up.

As the pattern of repeat and cross-over procedures is likely to be multiple and complex, these will be measured as outcomes and no attempt will be made to adjust for them. Effect sizes will be presented as point estimates, 95% confidence intervals, and associated *p* values.

### Sub-group analysis

Variation in the treatment effect between subgroups will be limited to prespecified variables and investigated using appropriate tests for interaction in survival and repeated measures models. Variables likely to be considered will include, but will not necessarily be restricted to, ischaemic rest/night pain only versus tissue loss, or both rest pain and tissue loss, presence of DM, and presence of CKD.

### Economic analysis

The economic analysis will comprise both ‘within-study’ and ‘model-based’ analyses. The ‘within-study’ analysis will be carried out to determine the cost-effectiveness of all three trial-mandated interventions on the basis of the patient-level data obtained during the study period.

Costs will take into account the use of NHS resources related to the primary interventions and any secondary procedures, readmissions, SAEs, hospital stays, day-case admissions, outpatient visits, and appointments with general practitioners and nurses. Changes in health-related QoL and QALYs will be calculated according to patients’ responses to the EQ-5D-5L instrument. Results of the analysis will be presented in terms of cost per year of AFS and cost per additional QALY gained. In line with existing recommendations, the base-case analysis will adopt a health care system (payer’s) perspective by considering costs incurred by the NHS and Personal Social Services [20].

The model-based analysis will be conducted to extend the within-study evaluation by considering costs and benefits likely to accrue beyond the study follow-up period. This will be based on a decision analytic model which will be built to serve as a framework for quantifying long-term costs and outcomes.

### Dissemination policy

The chief investigator will coordinate dissemination of data from BASIL-3. All publications and presentations, including abstracts, relating to the main trial will be authorised by the BASIL-3 TMG. The results of the analyses will be published in the name of the BASIL-3 Collaborative Group in peer-reviewed journals (provided that this does not conflict with the journal’s policy). All contributors to the trial will be listed, with their contribution identified. Trial participants will be sent a summary of the final results of the trial which will contain a reference to the full papers. All applications from groups wanting to use BASIL-3 data to undertake original analyses will be submitted to the TMG for consideration before release. To safeguard the scientific integrity of BASIL-3, trial data will not be presented in public before the main results are published without the prior consent of the TMG.

## Discussion

BASIL-3 is a pragmatic RCT which is designed to inform future UK ‘standard of care’ for patients presenting with SLI secondary to FP disease. Many of these patients will have multilevel disease including both ‘inflow’ (aorto-iliac) disease as well as ‘outflow’ or ‘below the knee’ (IP) disease. In such patients it will be permissible to perform additional interventions in accordance with routine practice at the same time as the randomised FP intervention. Moreover, it would also be permissible to restore inflow by performing an aorto-iliac intervention during an initial procedure and then randomise for an endovascular FP intervention at a later date if this is still deemed to be appropriate.

Enrolment into the BASIL-3 trial is on a per-patient basis and only one leg may be randomised into the trial. However, data from the original BASIL trial suggest that up to a quarter of patients may present with bilateral SLI, in which case the ‘worst’ leg (which is usually clinically obvious) should be entered as the trial limb.

### Trial status

At the time of writing, the BASIL-3 trial was halfway through the study’s pilot phase with 15 clinical centres open to recruitment and over 50 participants randomised.

## Additional file

Additional file 1: BASIL-3 SPIRIT Checklist. (DOC 122 kb)

## Abbreviations

ABPI: Ankle-brachial pressure index
AE: Adverse event
AFS: Amputation-free survival
BCTU: Birmingham Clinical Trials Unit
BMS: Bare metal stent
CKD: Chronic kidney disease
CRF: Case Report Form
DCB: Drug-coated balloon
DES: Drug-eluting stent
DM: Diabetes mellitus
DMC: Data Monitoring Committee
DMC: Data Monitoring Committee
FP: Femoro-popliteal
HTA: Health Technology Assessment Programme
IP: Infrapopliteal
ITT: Intention-to-treat
NICE: National Institute for Health and Care Excellence
NIHR: National Institute for Health Research
PAD: Peripheral arterial disease
PBA: Plain balloon angioplasty
PEDIS: Perfusion Extent Depth Ischaemia Sensation tool
QALY: Quality-adjusted life year
QoL: Quality of life
RCT: Randomised controlled trial
SAE: Serious adverse event
SLI: Severe limb ischaemia
TBPI: Toe-brachial pressure index
TSC: Trial Steering Committee
UK: United Kingdom
WIFI: Wound, Ischaemia, and Foot Infection tool
